# Protopanaxadiol-Enriched Rice Exerted Antiadipogenic Activity during 3T3-L1 Differentiation and Anti-Inflammatory Activity in 3T3-L1 Adipocytes

**DOI:** 10.3390/pharmaceutics15082123

**Published:** 2023-08-11

**Authors:** Chaiwat Monmai, Jin-Suk Kim, Hyun Bo Sim, Doh-Won Yun, Sung-Dug Oh, Eui-Shik Rha, Jong-Jin Kim, So-Hyeon Baek

**Affiliations:** 1 Department of Agricultural Life Science, Sunchon National University, Sunchon 59722, Republic of Korea; bbuayy@gmail.com (C.M.); kimjs6911@naver.com (J.-S.K.); euishik@scnu.ac.kr (E.-S.R.); 2 Department of Biomedical Science, Sunchon National University, Sunchon 57922, Republic of Korea; kokonun3@naver.com; 3 National Institute of Agricultural Sciences, Rural Development Administration, Jeonju 54874, Republic of Korea; dwyun@korea.kr (D.-W.Y.); ohbaboh@korea.kr (S.-D.O.)

**Keywords:** transgenic rice, protopanaxadiol, adipogenesis, adipocyte, PPARγ, C/EBPα

## Abstract

Ginseng is a traditional medicine with health benefits for humans. Protopanaxadiol (PPD) is an important bioactive compound found in ginseng. Transgenic rice containing PPD has been generated previously. In the present study, extracts of this transgenic rice were evaluated to assess their antiadipogenic and anti-inflammatory activities. During adipogenesis, cells were treated with transgenic rice seed extracts. The results revealed that the concentrations of the rice seed extracts tested in this study did not affect cell viability at 3 days post-treatment. However, the rice seed extracts significantly reduced the accumulation of lipids in cells and suppressed the activation of CCAAT/enhancer-binding protein α (C/EBPα) and peroxisome proliferator-activated receptor γ (PPARγ), which in turn inhibited the expression of adipogenesis-related mRNAs, such as adiponectin, PPARγ, C/EBPα, sterol regulatory element-binding protein 1, glucose transport member 4, and fatty acid synthase. In adipocytes, the extracts significantly reduced the mRNA expression of inflammation-related factors following LPS treatment. The activation of NF-κB p65 and ERK 1/2 was inhibited in extract-treated adipocytes. Moreover, treatment with extract #8 markedly reduced the cell population of the G2/M phase. Collectively, these results indicate that transgenic rice containing PPD may act as an obesity-reducing and/or -preventing agent.

## 1. Introduction

Excess energy can be stored in the form of lipids in adipocytes; however, the excessive accumulation of fat in the body is characterized as obesity, which is a major medical problem [1,2] and a serious global health concern [3]. Obesity contributes to several chronic diseases [4] and increases the risk of diabetes [5], cardiovascular disease [6], prostate disease [7], and respiratory disease [8].

Preadipocyte differentiation (adipogenesis) is the process through which mature adipocytes are formed; these adipocytes contribute to intracellular lipid accumulation [9]. During differentiation, several transcription factors, including CCAAT/enhancer-binding protein (C/EBP) and peroxisome proliferator-activated receptor γ (PPARγ), are regulated [10]. Sterol regulatory element-binding protein-1 (SREBP-1) is a transcription factor associated with adipocyte differentiation [11]. The activation of these transcription factors leads to the production of adipogenesis-related biomolecules, such as adiponectin, leptin [12], glucose transport member 4 (Glut4) [13], fatty acid synthase (FAS), acetyl-CoA carboxylase, and stearoyl-CoA desaturase-1 [14].

Ginseng (*Panax ginseng*) is used as a natural medicinal herb in the Republic of Korea, China, Japan, and several other counties [15]. Various studies have demonstrated the health benefits of ginseng in relation to immune function (both immune enhancement and anti-inflammatory activity) [16,17], cardiovascular disease [18,19], obesity [20,21], and type 2 diabetes [22]. Among the active compounds isolated from ginseng, ginsenosides are the main components with pharmaceutical potential [15]. Protopanaxadiol (PPD) is a major component of ginsenosides and exerts immune-associated effects, including anticancer effects caused by the inhibition of breast cancer proliferation [23], anti-inflammatory effects, and antistress effects in immobilized mice [24]. It can be used to enhance macrophage activities in innate immune responses [25] and inhibit 3T3-L1 and human protein atlas cell differentiation by reducing the protein and mRNA expression of PPARγ and C/EBPα [26].

In 2019, we introduced selected genes into Dongjin rice to generate PPD-producing rice [27]. In the generated transgenic rice, the expression of the selected genes was confirmed in T_1_ leaves and T_2_ seeds using genomic polymerase chain reaction (PCR) and qPCR, respectively. Furthermore, the amount of PPD in T_4_ transgenic rice seeds was quantified using liquid chromatography–mass spectrometry [28]. PPD-enriched rice exhibits potential health benefits, i.e., it increases the immune response and decreases inflammation biomarkers in LPS-stimulated RAW 264.7 macrophages [28,29], thereby exerting antioxidant activity, suppressing melanogenesis, and controlling hyperpigmentation in Melan-A melanocytes [30]. In the present study, transgenic rice seed extracts were investigated for their antiadipogenic activities during 3T3-L1 preadipocyte differentiation and anti-inflammatory activities in adipocytes.

## 2. Materials and Methods

### 2.1. Treatment Preparation

The treatments examined were the same as those used in our previous report [28], and the PPD content in each extract is shown in Table 1.

### 2.2. 3T3-L1 Cell Culture

The 3T3-L1 cells were kindly gifted by Prof. Young-Jin Son (Department of Pharmacy, Sunchon National University, Republic of Korea) and maintained in DMEM (Welgene, Gyeongsangbuk, Republic of Korea) containing 10% bovine calf serum (Welgene) and 100 µg/mL of penicillin/streptomycin (Hyclone, Logan, UT, USA). Cells were grown to confluence at 37 °C with 5% CO_2_.

### 2.3. Preadipocyte Differentiation

**Figure 1:**
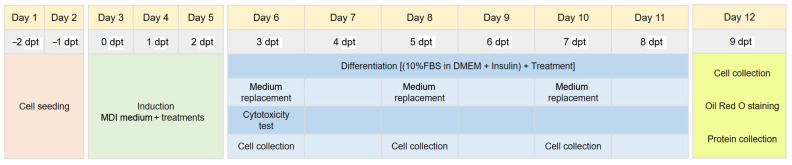
Overview of the experimental procedure and the timeline of adipogenesis (dpt, days post-treatment). Cells were collected for RNA extraction at 3, 5, 7, and 9 dpt (cell collection).

Differentiation was induced using MDI medium (1 µM dexamethasone [Sigma-Aldrich, Saint Louis, MO, USA], 500 µM 3-isobutyl-1-methylxanthine [IBMX; Sigma-Aldrich] and 10 µg/mL insulin [Sigma-Aldrich] in DMEM, supplemented with 10% fetal bovine serum [FBS: Gibco™, Thermo Fisher Scientific, Inc., Waltham, MA, USA]), at 0 days post-treatment (dpt). At 3 dpt, an adipogenic medium (DMEM supplemented with 10% FBS and 10 µg/mL insulin) was applied to the cells. These were then maintained until 9 dpt; the adipogenic medium was changed every 2 days. In addition, the cells were treated during the differentiation process. The experimental procedure is summarized in Figure 1. Adipocyte differentiation was monitored using an IM-3 series microscope (Optika, Ponteranica, Italy).

### 2.4. Cell Viability Assay

After differentiation for 3 days, the cells were collected for use in the analysis of the cytotoxic effects of extracts. Cell viability was analyzed using the 10% EZ-Cytox Cell Viability Assay Kit (DoGenBio, Seoul, Republic of Korea) in accordance with the manufacturer’s instructions.

### 2.5. Oil Red O Assay

The cells were fixed with 3.7% formalin (Sigma-Aldrich) at room temperature for 30 min. After discarding the formalin solution, the remaining solution was removed using distilled water. Subsequently, 100 μL of 60% isopropanol was added to the cells, which were then incubated at room temperature in the absence of light for 5 min. The cells were then stained with Oil Red O solution (0.3% (*v*/*v*), Sigma-Aldrich), incubated at room temperature (in the absence of light) for 15 min, washed with distilled water to remove the excess dye, and allowed to dry at room temperature by opening the plate cover. The stained lipid droplets were imaged under an IM-3 series microscope. The intracellular lipid content was measured by eluting the dye with 60% isopropanol using a SpectraMax^®^ ABS Plus (Molecular Devices, San Jose, CA, USA) at 500 nm. Intracellular lipid accumulation was calculated in reference to the medium group as follows:

(1) $$\mathrm{Lipid}\;\mathrm{accumulation}\;\left(\%\right)=\frac{A_{500}\;\mathrm{of}\;\mathrm{the}\;\mathrm{test}\;\mathrm{group}-A_{500}\;\mathrm{of}\;\mathrm{the}\;\mathrm{blank}}{A_{500}\;\mathrm{of}\;\mathrm{the}\;\mathrm{control}-A_{500}\;\mathrm{of}\;\mathrm{the}\;\mathrm{blank}}\;\times \;100$$

where A_500_ is the absorbance at 500 nm.

### 2.6. RNA Extraction and Real-Time PCR Analysis

Differentiated 3T3-L1 cells were collected at 3, 5, 7, and 9 dpt and washed with ice-cold 1× PBS. Total RNA was isolated using TRI Reagent™ solution (Invitrogen, Waltham, MA, USA). The RNA pellet was dissolved in nuclease-free water and kept at −80 °C. The concentration and purity of the extracted RNA were measured using a SpectraDrop™ microvolume microplate (Molecular Devices). cDNA was synthesized from 1 µg of total RNA using a Power cDNA Synthesis Kit (iNtRON Biotechnology, Inc., Gyeonggi, Republic of Korea). The PCR reactions and conditions used were similar to those described in a previous study [29]. The specific primer sets used in this study are shown in Table S1; *β-actin* was used as the reference gene.

### 2.7. Western Blotting Analysis

Western blotting analysis was performed according to the method of Monmai et al. [28]. The membranes were incubated at 4 °C overnight with the following antibodies: PPARγ (Santa Cruz Biotechnology, Dallas, TX, USA), C/EBPα (Santa Cruz Biotechnology), phosphorylated (p)-ERK 1/2 (Cell Signaling Technology, Danvers, MA, USA), ERK 1/2 (Cell Signaling Technology), p-Akt (Cell Signaling Technology), and Akt (Santa Cruz Biotechnology). Glyceraldehyde 3-phosphate dehydrogenase (GAPDH; Santa Cruz Biotechnology) was used as the protein loading control. Protein signaling was detected using Clarity Max™ Western ECL Substrate (Bio-Rad, Hercules, CA, USA). ChemiDoc Imaging System (Bio-Rad) was used for detecting, imaging, and quantifying signals in terms of intensity. Protein expression was presented in terms of an expression ratio to the medium group (% fold).

### 2.8. 3T3-L1 Cell Differentiation and LPS-Stimulated Adipocyte Viability Assay

**Figure 2:**
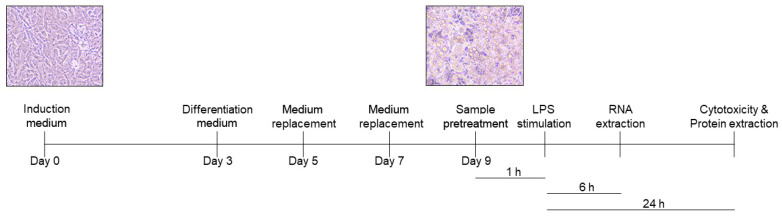
Overview of the anti-inflammatory experimental procedure in LPS-stimulated adipocytes.

The anti-inflammatory effects of rice seed extracts in 3T3-L1 adipocytes were measured, as shown in Figure 2, in the absence of treatment. On day 9, adipocytes were treated with treatments prepared in a cell culture medium. At 1 h after treatment, the cells were stimulated with 1 µg/mL LPS. Cell viability was determined as described in Section 2.4.

### 2.9. Real-Time PCR and Western Blotting in LPS-Stimulated Adipocytes

Total RNA and protein were extracted from LPS-stimulated adipocytes at 6 and 24 h after LPS stimulation, respectively, using the method described in Section 2.6. The sequences of the primer sets used are shown in Table S1. Western blotting was performed as described in Section 2.7. The specific antibodies used for western blotting were p-ERK 1/2 (Cell Signaling Technology), ERK 1/2 (Cell Signaling Technology), p-NF-κB p65 (Cell Signaling Technology), NF-κB p65 (Santa Cruz Biotechnology), and GAPDH (Santa Cruz Biotechnology).

### 2.10. Cell Cycle Assay

The cells were treated with MDI and rice seed extract #8 (100 µg/mL) in 48-well plates (6 × 10^4^ cells/well). After 20 h, the cells were harvested in a 5 mL tube and stained with propidium iodide (PI). In brief, the cells were washed with PBS and fixed with 70% EtOH at 4 °C for 2 h. Then, the fixed cells were washed with cold PBS and stained using the FxCycle^TM^ PI/RNase staining solution (Invitrogen, MA, USA). The stained cells were quantified via flow cytometry (FACS canto II, BD Biosciences, San Jose, CA, USA). Data analyses were conducted using FlowJo software version 10.9.0.

### 2.11. Statistical Analysis

Experimental data are presented as mean ± standard deviation. Statistix8.1 software (Statistix, Tallahassee, FL, USA) was used for statistical analysis. Significant differences were determined using one-way analysis of variance (ANOVA), followed by the application of Duncan’s multiple-range tests. Differences were considered significant if *p* < 0.05.

Statistical analysis of the cell cycle was performed using SPSS ver. 22 (SPSS, Chicago, IL, USA). Student’s *t* test was used to make comparisons between the two groups, and one-way ANOVA with Duncan’s multiple-range test was used to make comparisons among more than two groups.

## 3. Results

### 3.1. Effects of PPD-Containing Rice Seed Extracts on Cell Viability

**Figure 3:**
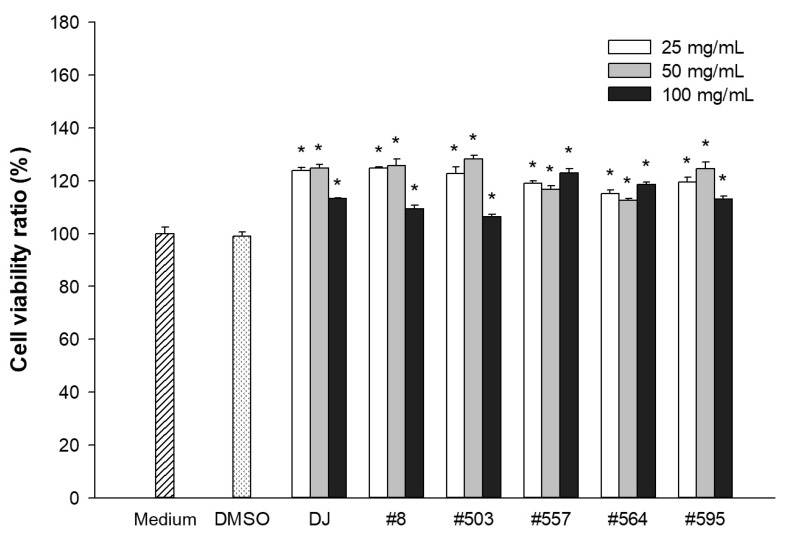
Effects of PPD-containing rice seed extracts on the viability of 3T3-L1 cells. Medium: untreated group (MDI medium); DMSO: 0.1% DMSO; DJ: normal rice seed extract; #8: transgenic rice line 8 seed extract; #503: transgenic rice line 503 seed extract; #557: transgenic rice line 557 seed extract; #564: transgenic rice line 564 seed extract; #595: transgenic rice line 595 seed extract. Data are presented as mean ± standard deviation. * Significant difference at *p* < 0.05 vs. DMSO.

The toxicity exerted by PPD-containing rice seed extracts on 3T3-L1 cells was evaluated at 3 dpt. The cells were incubated with the EZ-Cytox Cell Viability Assay Kit (DoGenBio, Seoul, Republic of Korea) in accordance with the manufacturer’s instructions. An MDI medium served as the control in the untreated group (medium). Treatment with 0.1% dimethyl sulfoxide (DMSO) resulted in cell viability equivalent to that of untreated cells (Figure 3). Compared with treatment with the medium, all concentrations of transgenic rice seed extracts and normal rice seed extracts (DJ) significantly enhanced cell proliferation. PPD-containing rice seed extracts at concentrations up to 100 µg/mL had no negative effects on cell viability.

### 3.2. Effects of PPD-Containing Rice Seed Extracts on Lipid Accumulation

**Figure 4:**
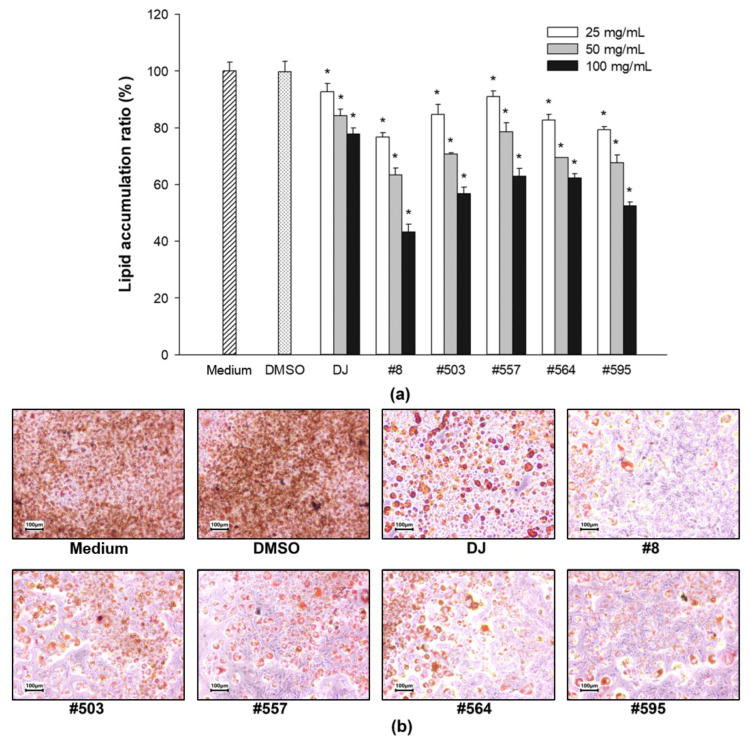
Effects of PPD-containing rice seed extracts on lipid accumulation in 3T3-L1 cells. (**a**) Lipid accumulation ratio following various treatments. Medium: untreated group (adipogenic medium); DMSO: 0.1% DMSO; DJ: normal rice seed extract; #8: transgenic rice line 8 seed extract; #503: transgenic rice line 503 seed extract; #557: transgenic rice line 557 seed extract; #564: transgenic rice line 564 seed extract; #595: transgenic rice line 595 seed extract. (**b**) Representative images of Oil Red O staining of each treatment at the concentration of 100 µg/mL. Data are presented as mean ± standard deviation. * Significant difference at *p* < 0.05 vs. DMSO.

Cells treated with the adipogenic medium were assigned to the positive control group (medium). Lipid accumulation in 0.1% DMSO-treated cells was similar to that in medium-treated cells (Figure 4). However, treatment with either normal (DJ) or transgenic rice seed extracts at the lowest concentration (25 µg/mL) significantly reduced lipid accumulation, and lipid accumulation gradually decreased further as the extract concentration increased. Lipid accumulation in cells treated with 100 µg/mL of extracts #8, #503, #557, #564, and #595 was markedly lower than that in cells treated with the DJ extract. The PPD content in the seeds was significantly correlated with the reduction in lipid accumulation in the cells (Pearson’s correlation coefficient = 0.752). Therefore, PPD-containing rice seed extracts appeared to suppress adipogenesis by reducing lipid accumulation in a dose-dependent manner.

### 3.3. Effects of PPD-Containing Rice Seed Extracts on Cell Differentiation

**Figure 5:**
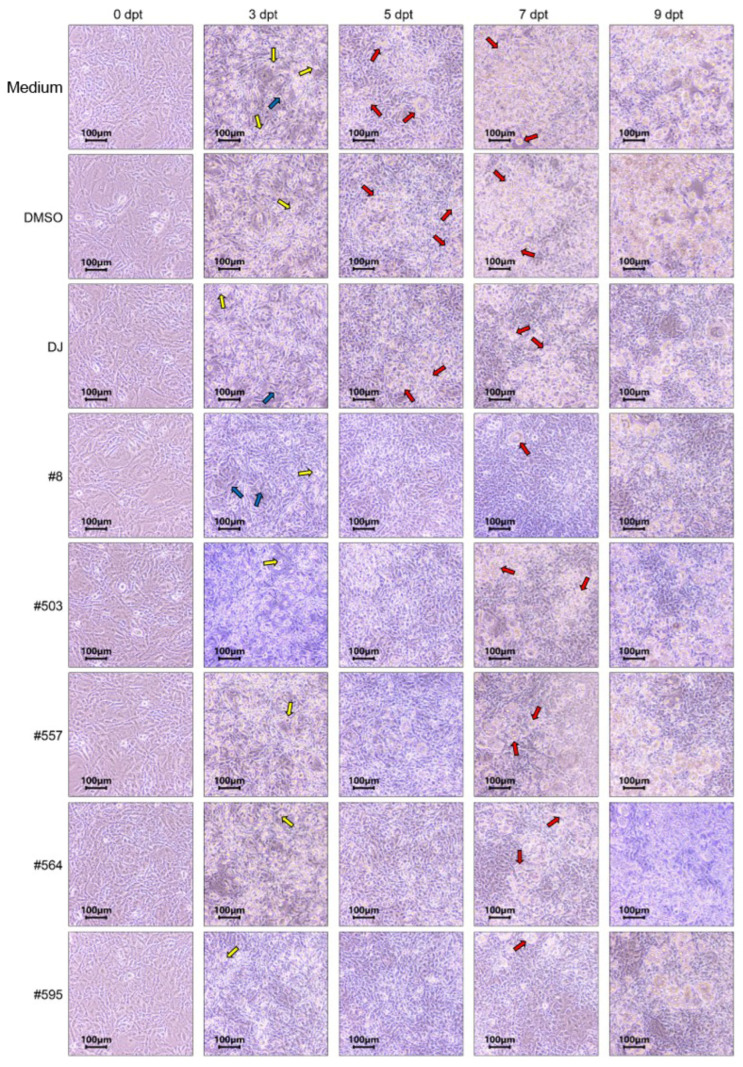
Representative images of 3T3-L1 cells from induction (Day 0) to 9 days post-induction (Day 9). Medium: untreated group (adipogenic medium); DMSO: 0.1% DMSO; DJ: normal rice seed extract; #8: transgenic rice line 8 seed extract; #503: transgenic rice line 503 seed extract; #557: transgenic rice line 557 seed extract; #564: transgenic rice line 564 seed extract; #595: transgenic rice line 595 seed extract. Blue arrows represent the fibroblast-like cell shape during the differentiation process. Yellow arrows represent the round shape of cells. Red arrows represent the lipid-containing cells.

After preadipocytes reached confluence (designated as 0 dpt), change was chemically induced in the cells to obtain mature adipocytes. Before the induction of differentiation using MDI medium, the preadipocytes appeared as fibroblasts. At 3 dpt, the cells were visibly rounder. A small number of lipid droplets was detected in medium-, DMSO-, and DJ-treated cells at 5 dpt, and lipid droplets were clearly visible at 7 dpt (Figure 5). However, lipid accumulation was slightly delayed due to treatment with PPD-producing rice seed extracts, i.e., it was clearly visible at 7 dpt.

### 3.4. Effects of PPD-Containing Rice Seed Extracts on Adipogenic Gene Expression

**Figure 6:**
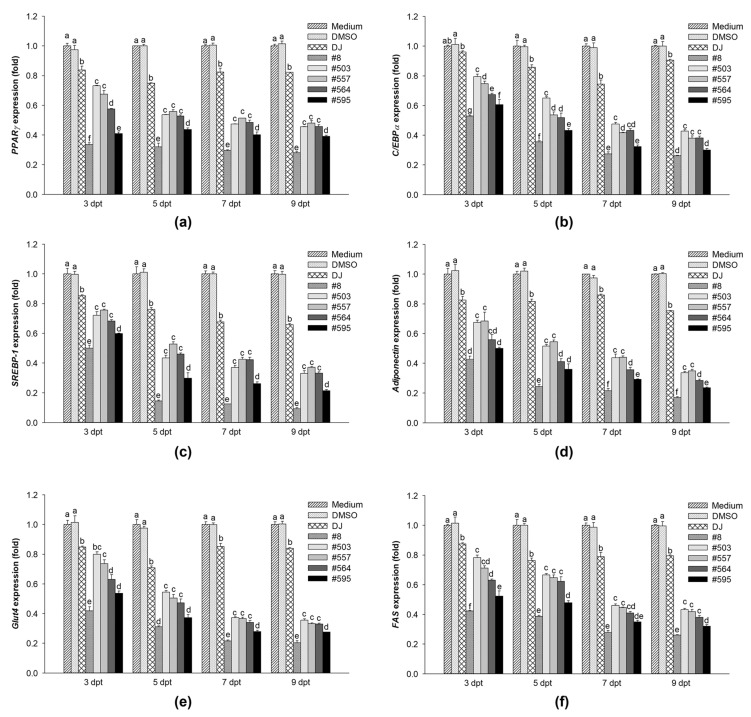
Effects of PPD-containing rice seed extracts (100 µg/mL) on adipogenic-related mRNA expression levels. Expression of (**a**) *PPARγ*, (**b**) *C/EBPα*, (**c**) *SREBP-1*, (**d**) adiponectin, (**e**) *Glut4*, and (**f**) *FAS* mRNA. Medium: untreated group; DMSO: 0.1% DMSO; DJ: normal rice seed extract; #8: transgenic rice line 8 seed extract; #503: transgenic rice line 503 seed extract; #557: transgenic rice line 557 seed extract; #564: transgenic rice line 564 seed extract; #595: transgenic rice line 595 seed extract. Data are presented as mean ± standard deviation. Different letters (a–g) indicate significant differences at *p* < 0.05 among the medium, DMSO, DJ, #8, #503, #557, #564, and #595 groups on the same differentiation day.

The expression levels of *PPARγ*, *SREBP-1*, and *C/EBPα* were evaluated. Treatment with 100 µg/mL of rice seed extracts (either normal or transgenic rice) markedly decreased MDI-induced *PPARγ*, *C/EBPα*, and *SREBP-1* mRNA expression in the early stage of adipogenesis (3 dpt), and the expression remained suppressed at 9 dpt (Figure 6a–c). Because the expression of transcription factors was reduced, the expression of adipocyte markers and fat-forming genes was also reduced (adiponectin, *Glut4*, and *FAS*) (Figure 6d–f). Compared with DJ, treatment with PPD-containing rice seed extracts had the potential to reduce the mRNA expression of adipogenesis-related transcription factors and biomarkers from 3 to 9 dpt. In particular, 100 µg/mL of extract #8 (with the highest PPD content) suppressed the expression of adipogenic transcription factor genes, adiponectin, *Glut4*, and *FAS*, most potently out of those examined.

### 3.5. Effects of PPD-Containing Rice Seed Extracts on Protein Expression

**Figure 7:**
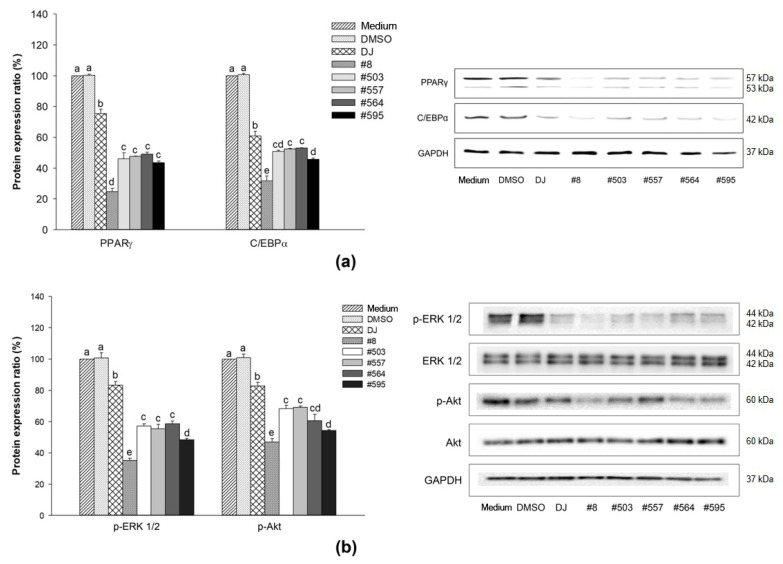
Effects of PPD-containing rice seed extracts on protein expression in adipocytes. (**a**) Adipogenic proteins (PPARγ and C/EBPα) and (**b**) p-ERK 1/2 and p-Akt proteins. Medium: untreated group (adipogenic medium); DMSO: 0.1% DMSO; DJ: normal rice seed extract; #8: transgenic rice line 8 seed extract; #503: transgenic rice line 503 seed extract; #557: transgenic rice line 557 seed extract; #564: transgenic rice line 564 seed extract; #595: transgenic rice line 595 seed extract. Lowercase letters (a–e) indicate significant differences at *p* < 0.05 among the medium, DMSO, DJ, #8, #503, #557, #564, and #595 groups.

The protein expression of PPARγ and C/EBPα was upregulated in adipogenic-induced cells (medium group) and 0.1% DMSO-treated cells (Figure 7a). Conversely, treatment with either normal (DJ) or transgenic rice seed extracts significantly reduced the protein expression of PPARγ and C/EBPα. Furthermore, the protein expression of PPARγ and C/EBPα in cells treated with PPD-containing rice seed extracts was markedly lower than that seen in cells treated with normal rice. The cells treated with extract #8 had the lowest PPARγ expression (75.24% and 67.14% decreases compared with the medium and DJ groups, respectively) and the lowest C/EBPα expression (68.30% and 48.00% decreases compared with the medium and DJ groups, respectively).

Cells treated with PPD-containing rice seed extracts had markedly suppressed levels of phosphorylated(p)-ERK 1/2, and p-Akt (Figure 7b). The largest downregulation of p-ERK 1/2 and p-Akt protein expression was observed following treatment with extract #8, which had the highest PPD content.

### 3.6. Effects of PPD-Containing Rice Seed Extracts on the Viability of LPS-Stimulated Adipocytes

**Figure 8:**
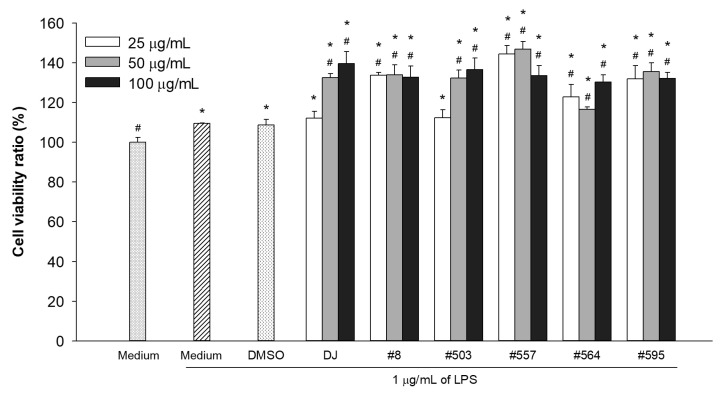
Effects of PPD-containing rice seed extracts on the cell viability of LPS-stimulated adipocytes. Medium: untreated group (DMEM); DMSO: 0.1% DMSO; DJ: normal rice seed extract; #8: transgenic rice line 8 seed extract; #503: transgenic rice line 503 seed extracts; #557: transgenic rice line 557 seed extracts; #564: transgenic rice line 564 seed extract; #595: transgenic rice line 595 seed extract. Data are presented as mean ± standard deviation. Significant differences at *p* < 0.05 were determined compared with the medium (no LPS stimulation; *) and DMSO (#) groups.

As shown in Figure 8, treatment with 1 µg/mL LPS significantly increased cell proliferation compared with the medium-treated group (*p* < 0.05). However, no significant difference (*p* < 0.05) was observed in terms of the cell viability of LPS-stimulated adipocytes between the medium and DMSO groups. Treatment with rice seed extracts (100 µg/mL) markedly enhanced the proliferation of LPS-stimulated adipocytes compared with the DMSO group (*p* < 0.05). Therefore, a concentration of 100 µg/mL was selected for the subsequent experiments.

### 3.7. Effects of PPD-Containing Rice Seed Extracts on Immune-Related mRNA in LPS-Stimulated Adipocytes

**Figure 9:**
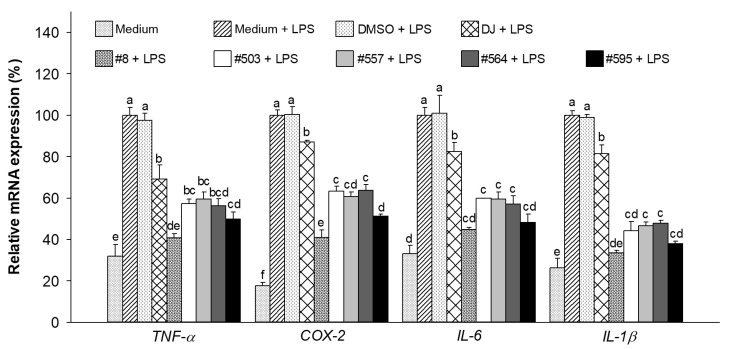
Effects of PPD-containing rice seed extracts on inflammation-related mRNA expression levels in LPS-stimulated adipocytes. Medium: untreated group (DMEM); DMSO: 0.1% DMSO; DJ: normal rice seed extract; #8: transgenic rice line 8 seed extract; #503: transgenic rice line 503 seed extract; #557: transgenic rice line 557 seed extract; #564: transgenic rice line 564 seed extract; #595: transgenic rice line 595 seed extract. Data are presented as mean ± standard deviation. Difference letters (a–f) indicate significant differences at *p* < 0.05 among the medium, DMSO, DJ, #8, #503, #557, #564, and #595 groups.

Treatment with LPS significantly increased the expression level of inflammation-involved genes in medium-treated cells (Figure 9). The pretreatment of cells with PPD-enriched rice seed extracts (100 µg/mL) caused a statistically significant decrease in the expression levels of LPS-induced inflammatory genes. The mRNA expression of *TNF-α*, *COX-2*, *IL-6*, and *IL-1β* was reduced by 59.19%, 59.06%, 55.20%, and 66.37%, respectively, in the cells treated with #8 and LPS compared with the level seen in cells treated with LPS treatment alone.

### 3.8. Effects of PPD-Containing Rice Seed Extracts on the Activation of Immune-Related Pathways in LPS-Stimulated Adipocytes

**Figure 10:**
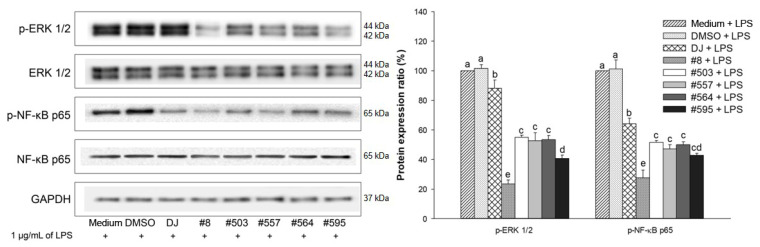
Effects of 100 µg/mL of PPD-containing rice seed extracts on the protein expression of p-ERK 1/2 and p-NF-κB p65 in LPS-stimulated adipocytes. Medium: untreated group (DMEM); DMSO: 0.1% DMSO; DJ: normal rice seed extract; #8: transgenic rice line 8 seed extract; #503: transgenic rice line 503 seed extract; #557: transgenic rice line 557 seed extract; #564: transgenic rice line 564 seed extract; #595: transgenic rice line 595 seed extract. Data are presented as mean ± standard deviation. Different letters (a–e) indicate significant differences at *p* < 0.05 among the medium, DMSO, DJ, #8, #503, #557, #564, and #595 groups.

The expression of p-ERK 1/2 and p-NF-κB p65 was upregulated in LPS-treated adipocytes (medium group) (Figure 10). Cotreatment with 100 µg/mL of PPD-enriched rice seed extracts and 1 µg/mL of LPS significantly downregulated the protein expression of p-ERK 1/2 and p-NF-κB p65 compared with the use of the normal rice group (DJ). LPS-induced ERK 1/2 and NF-κB p65 expression was the most suppressed in extract #8-treated cells. Compared with DJ, treatment with #8 significantly reduced the expression level of p-ERK 1/2 from 88.23% ± 5.56% to 23.50% ± 2.63% and that of p-NF-κB p65 from 64.28% ± 3.62% to 27.64% ± 5.13% (*p* < 0.05).

### 3.9. Effects of PPD-Containing Rice Seed Extracts on Cell Cycle

**Figure 11:**
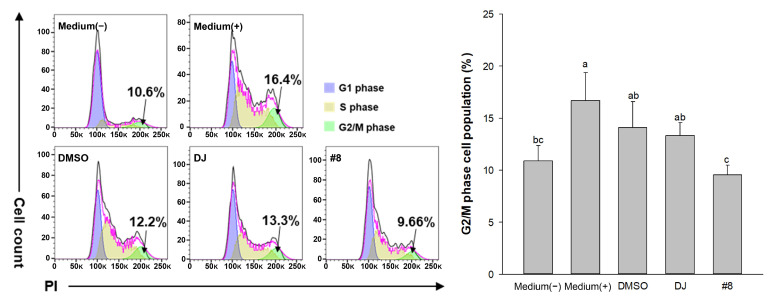
Assessment of cell cycle. The cell cycle was analyzed via flow cytometry. Data are presented as mean ± standard deviation. Medium (−): untreated group (normal medium); Medium (+): untreated group (adipogenic medium); DMSO: 0.1% DMSO; DJ: normal rice seed extract; #8: transgenic rice line 8 seed extract. Lowercase letters (a–c) indicate significant differences at *p* < 0.05 among the medium (−), medium (+), DMSO, DJ, and #8.

Differentiated adipocytes display cell cycle inhibition in the G2/M phase [31]. Collectively, the data indicated that extract #8 exhibited the strongest antiadipogenic and anti-inflammatory activities. The population of cells in the G2/M phase was evaluated in cells treated with #8. We checked each cell cycle phase, i.e., G1, S, and G2/M, by performing staining with PI. MDI-treated differentiated cells had a high proportion of cells in the G2/M phase, as expected (Figure 11). Interestingly, the induction of the G2/M phase was inhibited by treatment with PPD-producing rice seed extracts, especially extract #8. Important factors related to the induction of adipocyte differentiation, such as cell cycle progression to the G2/M phase, were clearly regulated by PPD-producing transgenic rice seed extract #8.

## 4. Discussion

The antiadipogenic activities of PPD-containing rice seed extracts were evaluated in 3T3-L1 cells. The cytotoxicity of PPD-containing rice seed extracts in 3T3-L1 cells was assessed to determine a suitable concentration for use in our antiadipogenic investigation. Low concentrations (25 and 50 µg/mL) of PPD-containing rice seed extracts promoted cell proliferation (Figure 3), whereas high concentrations (150 and 250 µg/mL) reduced cell viability. Therefore, the antiadipogenic effects of PPD-containing rice seed extracts were assessed using extract concentrations up to 100 µg/mL.

Cotreatment with IBMX and dexamethasone induces the differentiation of preadipocytes by regulating the expression of PPARγ [32]. The expression of PPARγ is induced by insulin in the final stage of adipogenesis [33]. Rosen et al. [34] reported that PPARγ promoted adipogenesis when combined with C/EBPα; however, adipogenesis was not induced in C/EBPα-deficient cells in the absence of PPARγ. In the present study, the expression of PPARγ and C/EBPα (mRNA and protein) was promoted in preadipocytes cultured with MDI medium (Figure 6a,b and Figure 7), whereas treatment with PPD-producing rice seed extracts significantly reduced the expression levels of PPARγ and C/EBPα. These findings are consistent with those of Zhang et al. [35], who found that the expression of the PPARγ and C/EBPα proteins was suppressed by heated PPD ginsenoside (ginsenoside Rg3). In the present study, the suppression of PPARγ and C/EBPα protein expression affected the accumulation of lipid droplets in the cells, as shown in Figure 7a and Figure 4. Similarly, in a previous study, one of the PPD ginsenosides (ginsenoside Rc) was found to decrease lipid accumulation in cells by suppressing PPARγ and C/EBPα expression [36].

The treatment of cells with PPD-containing rice seed extracts also led to the regulation of the ERK 1/2 pathway (Figure 7b), which is an important pathway for adipogenesis [37]. Our results showed that the expression levels of p-ERK 1/2 were significantly suppressed in the cells treated with PPD-containing rice seed extracts compared with the normal rice group (DJ). These results were similar to those of Belmonte et al. [38], who suggested that the activation of PPARγ and C/EBPα is associated with p-ERK 1/2. Prusty et al. [39] reported that the activation of ERK 1/2 enhanced adipogenesis by increasing the expression of PPARγ and C/EBPα. In addition, Donzelli et al. [40] highlighted the importance of ERK 1/2 in the adipogenic differentiation of human mesenchymal stem cells. In addition to p-ERK 1/2 (the MAPK pathway), Akt induces adipocyte differentiation [41]. As shown in Figure 7b, the activation of Akt (p-Akt) was significantly decreased in the PPD-producing rice seed extract treatment groups, similar to the findings of Li et al. [42]. Li et al. [42] reported that their sample exhibited antiadipogenic activity via the downregulation of major adipogenic differentiation-related genes and Akt protein expression.

Adipokines and adipocytokines are induced during the regulation of adipogenesis [43]. We demonstrated that the expression of *Glut4* and *Fas* was upregulated in PPARγ- and C/EBPα-activated cells. Adiponectin levels are known to be increased in differentiated adipocytes depending on the triglyceride content of the adipocytes [44,45]. We found that PPD-containing rice seed extracts inhibited the expression of adipogenic-related mRNAs, including adiponectin, *Glut4*, and *FAS* (Figure 6). These results were similar to those of Zhang et al. [35], who found that ginsenoside Rg3 suppressed the expression of adipogenic markers. Lee et al. [46] studied the in vitro and in vivo antiadipogenic effects of ginsenoside Rg3 and found that ginsenoside Rg3 downregulated the expression levels of adipogenesis-associated mRNA by suppressing the expression of PPARγ.

Obesity has been reported to induce inflammatory cytokine secretion in adipose tissue [47,48], leading to increased risks of type 2 diabetes, hypertension, metabolic syndrome, and hypertriglyceridemia [49]. The activation of the MAPK and NF-κB signaling pathways is the key to the regulation of acute inflammatory responses [50,51,52]. Our study demonstrated that the expression of p-ERK 1/2 (MAPK signaling pathway) and p-NF-κB p65 (NF-κB signaling pathway) was significantly suppressed by cotreatment with LPS and PPD-containing rice seed extracts (100 µg/mL). Further, we showed that this led to the inhibition of the mRNA expression of LPS-induced inflammatory cytokines. The expression of *IL-1β* and *IL-6*, which are involved in chronic inflammation associated with obesity [53], was also significantly decreased in cells treated with transgenic rice seed extracts.

Mitotic clonal expansion is one of the most relevant stages of adipocyte differentiation [54]. During the differentiation process, the cell population of the G0/G1 phase decreases, whereas the cell population of the G2/M phase increases [55]. Our result showed that treatment with extract #8 at 100 µg/mL markedly decreased the cell population of the G2/M phase (Figure 11). Thus, the content of PPD in rice seeds can be considered to cause the inhibition of adipogenesis factors during 3T3-L1 cell differentiation.

## 5. Conclusions

In summary, we investigated the antiadipogenic effects of PPD-enriched rice seed extracts on 3T3-L1 cells. The MDI-induced activation of PPARγ and C/EBPα transcription factors was inhibited by treatment with these extracts, and the suppression of these transcription factors led to reductions in several adipogenic markers, including lipid accumulation and the expression of *PPARγ*, *C/EBPα*, *SREBP-1*, adiponectin, *Glut4*, and *FAS*. In addition, transgenic rice seed extracts suppressed the LPS-induced activation of p-ERK 1/2 protein expression, p-NF-κB p65 protein expression, and proinflammatory cytokine production. Treatment with extract #8 significantly reduced the cell population of the G2/M phase in MDI-induced cells. The findings of this study indicate that transgenic rice containing PPD may be a potential obesity-reducing and/or -preventing agent.

## Figures and Tables

**Table 1:** PPD content of rice seed extracts.

Treatment	PPD Content (µg/g Dry Weight of Extract)
DJ	n.d.
#8	7.28 ± 0.64
#503	1.13 ± 0.03
#577	1.30 ± 0.03
#564	1.28 ± 0.08
#595	2.36 ± 0.07

Note: n.d.: not detectable.

## Data Availability

All applicable data have been provided in the manuscript. The authors will provide additional details if necessary.

## Supplementary Materials

The following supporting information can be downloaded at: https://www.mdpi.com/article/10.3390/pharmaceutics15082123/s1, Table S1: Sequences of the specific primers used for real-time PCR analysis.
