# Supplementary material for: Protopanaxadiol-Enriched Rice Exerted Antiadipogenic Activity during 3T3-L1 Differentiation and Anti-Inflammatory Activity in 3T3-L1 Adipocytes
Source: Pharmaceutics. 2023 Aug 11;15(8):2123. doi: 10.3390/pharmaceutics15082123 (PMC10458103; doi:10.3390/pharmaceutics15082123)
Supplement: Supplementary file 1 [file pharmaceutics-15-02123-s001.zip › pharmaceutics-2499542-supplementary.pdf]

Supplementary Materials

Table S1. Sequences of the specific primers used for real-time PCR analysis.

Target	Sequence (5′–3′)	Accession no.
PPARγ	Forward primer: CCCTGGCAAAGCATTTGTAT<br>Reverse primer: GAAACTGGCACCCTTGAAAA	AB644275.1
C/EBPα	Forward primer: TTACAACAGGCCAGGTTTCC<br>Reverse primer: AACTCCAGTCCCTCTGGGAT	NM_001287514.1
SREBP-1	Forward primer: AGCTCAAAGACCTGGTGGTG<br>Reverse primer: TCATGCCCTCCATAGACACA	BC056922.1
Adiponectin	Forward primer: AAAGGAGAGCCTGGAGAAGC<br>Reverse primer: GTAGAGTCCCGGAATGTTGC	NM_009605.5
Glut4	Forward primer: GCTGTTCCTTCAACCTGGAG<br>Reverse primer: GCAGGAGGTGAAACCCAGTA	NM_009204.2
FAS	Forward primer: CTCTGATCAGTGGCCTCCTC<br>Reverse primer: TGCTGCAGTTTGGTCTGAAC	AF127033.1
IL-1β	Forward primer: GGGCCTCAAAGGAAAGAATC<br>Reverse primer: TACCAGTTGGGGAACTCTGC	NM_008361.4
IL-6	Forward primer: AGTTGCCTTCTTGGGACTGA<br>Reverse primer: CAGAATTGCCATTGCACAAC	NM_031168.2
COX-2	Forward primer: AGAAGGAAATGGCTGCAGAA<br>Reverse primer: GCTCGGCTTCCAGTATTGAG	NM_011198.4
TNF-α	Forward primer: ATGAGCACAGAAAGCATGATC<br>Reverse primer: TACAGGCTTGTCACTCGAATT	D84199.2
β-actin	Forward primer: CCACAGCTGAGAGGGAAATC<br>Reverse primer: AAGGAAGGCTGGAAAAGAGC	NM_007393.5
